# Changes in Antioxidant Defense Capability and Lipid Profile after 12-Week Low- Intensity Continuous Training in Both Cigarette and Hookah Smokers: A Follow-Up Study

**DOI:** 10.1371/journal.pone.0130563

**Published:** 2015-06-29

**Authors:** Abdessalem Koubaa, Moez Triki, Hajer Trabelsi, Liwa Masmoudi, Zouhair Sahnoun, Ahmed Hakim

**Affiliations:** 1 Laboratory of Pharmacology, Sfax Medicine Faculty SMF, Avenue Majida Boulila, Sfax, 3029, Tunisia; 2 Research Unit (EM2S), Sfax Institute of Sport and Physical Education, Airport Road, P.O Box 384, Sfax, 3000, Tunisia; 3 Laboratory of cardio-circulatory, respiratory, and hormonal adaptations to muscular exercise, 98/UR08-67, Ibn El Jazzar Medicine Faculty, University of Sousse, Avenue Mohamed Karoui, Sousse, 4002, Tunisia; Temple University, UNITED STATES

## Abstract

To examine the impact of low-intensity continuous training program on antioxidant defense capability and lipid profile in male cigarette or hookah smokers. Forty-three male adults participated in a 12-week continuous training program at an intensity of 40% of VO_2_max. All subjects were subjected to anthropometric, physical and biochemical tests before and after the training program. The increase of Glutathione reductase (GR) and Superoxide dismutase (SOD) is significant only for cigarette smokers (CS) and hookah smokers (HS) groups. The Malondialdehyde (MDA) decrease and α-tocopherol increase are significant only for HS group. GPx was increased in NS, CS and HS by 2.6% (p< 0.01), 2% (p< 0.05) and 1.7% (p< 0.05) respectively. Likewise, significant improvements of high-density lipoprotein cholesterol (HDL-C), low-density lipoprotein cholesterol (LDL-C) and TC / HDL-C ratio were observed in three groups. En contrast no significant changes were recorded in triglycerides (TG). Also, significant reduction of total cholesterol (TC) for CS group (p< 0.01) and HS groups (p< 0.05). This continuous training program appears to have an important role in lipid levels improving and oxidative stress attenuation.

## Introduction

Smoking is the biggest public health threat of the current era. Worldwide, it is known as a risk factor for many diseases such as cardiovascular, respiratory, and cancer [1, 2]. In addition, sedentary lifestyle is considered a risk factor for several diseases [3] and oxidative stress is found linked to the development of several chronic diseases including atherosclerosis [4].

Oxidative stress describes a state of physiological stress in the body that arises from exposure to high levels of reactive oxygen species (ROS) to an extent that overwhelms the antioxidant defense system [5]. The oxidative damage of cell components has been implicated in the pathogenesis of a wide variety of diseases, most notably heart disease and cancer [6]. Recent studies suggest that oxidative stress induced by cigarette smoking poses a significant human health concern, especially as related to cardiovascular disease [7].

Cigarette smoking exacerbates ROS formation [8], evidenced by the increase in oxidative stress biomarkers in smokers compared with no smokers [9,10]. In this context, previous studies indicate that smokers have higher oxidative stress levels compared to nonsmokers, and this can be explained in part, by reduced blood antioxidant capacity [11,12]. Increased production of ROS from tobacco is recognized because of the more than 4,000 chemical substances found in tobacco [13]. Therefore, ill-health related to smoking may be linked to increased production of ROS.

Cigarette or hookah smoking induces almost the same causes of decreased levels of glutathione reductase (GR) and glutathione peroxidase (GPx), and α-tocopherol, unlike to nonsmoker subjects [14]. Moreover, as for cigarette smoking, hookah consumption involves significant increase in Low-density lipoprotein cholesterol (LDL-C) and is associated with high concentration of triglycerides (TG) and reduced concentrations of high-density lipoprotein cholesterol (HDL-C) [15,16]. In this context, Koubaa et al. study [14] consolidates the evidence that hookah consumption is associated with exposure to toxic substances and products, as those of cigarette smoking.

Anaerobic training has been shown to be beneficial in increasing antioxidants (SOD, GPx and GR) and reinforcement of human organism against incoming oxidative attacks [17,18]. Some studies have reported a decrease in antioxidant defense, which could be due to excessive production of free radicals by cumulative effect of anaerobic exercise [19,20]. Other studies have found that a regular physical activity could change the balance pro-oxidant / antioxidant and increase the endogenous antioxidants activity and the LDL resistance to oxidation. In contrast acute physical activity increases the consumption of oxygen and the production of free radicals and, therefore, may induce lipid peroxidation [21,22]. Within limits, it seems possible that anaerobic training can lead to attenuation of oxidative stress similar to aerobic training [17].

The antioxidant activity response to exercises is a subject of debate. Some experimental studies have demonstrated an increase [23,24], while others have noted no changes [25] or even a decrease [26]. These differences may be explained partly by diversity in training protocols, lifestyle or smoking habits and age of subjects.

Physical training has an important role in improving of lipid and lipoproteins levels, because dietary or calorie restriction alone has been shown to be an ineffective method of improving the lipids and lipoproteins profile [27]. Other studies suggest that regular aerobic exercise has been associated with favorable changes in lipid and lipoprotein levels [28,29]. Conversely, intensive physical training in presence of elevated serum lipids may even aggravate the atheromatosis development by releasing catecholamine that damage blood vessels, leading to lipid deposition [30].

In addition, few studies have generated evidence on the resistance training effects on lipid and lipoprotein levels. The results of the effects of this training mode are not consistent [31–34]. The findings of I Shaw and B. S. Shaw [35] demonstrated that resistance training has not been associated with favorable changes in the lipid and lipoprotein levels in sedentary male smokers. In contrast, Pate et al. [36] have proposed to do at least 30 minutes of physical activity of moderate intensity most days of the week. Likewise, previous studies recommended vigorous endurance exercises for at least 20 minutes three or more times a week [37].

These findings lead us to the idea of identifying the potential benefits of moderate physical activity in preventing smoking harms and explore the appropriate physical activity method for most smokers. Training benefits are optimized when programs are planned to meet the individual capacities of the participants. Therefore respiratory capacity must be taken into account in order to meet individual needs in training of sedentary smoker participants. The interest in assessing continuous training was based upon previous experiences of the acceptance of this training type in clinical practice [38,39], and because continuous training does not require an intense effort, smokers were encouraged to participate in this study.

Continuous exercise seems to be an important factor for improving cardiovascular function and quality of life in smoker participants. That may have important implications in antioxidant capacity and serum lipid concentrations.

Thus, the aim of this study is to investigate whether low-intensity continuous training could improve antioxidant enzymes activity and the lipids and lipoproteins profile of sedentary adults’ smokers, and check the difference of these individual training effects among cigarette smokers compared to hookah.

## Materials and Methods

### Participants

Our population was composed of adults matched in gender and age from the same ethnicity and socio-economic environment. In fact, forty-three sedentary and healthy male smokers and non smokers from the general community of Tunisia volunteered to participate in this study and were recruited within pharmacology laboratory of the Faculty of Medicine, university of Sfax, Tunisia. The anthropometric and physical characteristics of the participants are shown in Table 1.

**Table 1:** Anthropometric and physical characteristics of the participants.

Parameters	Means±SD			ANOVA
	NS	CS	HS	
**Age (yrs)**	43.8±2.1	43.2±2.1	43.7±2.3	F (2;40) = 0.2; p = 0.82; η_p_^2^ = 0.014
**Height (cm)**	175.6±2.2	175.9±1.5	175.3±1.5	F (2;40) = 0.32; p = 0.75; η_p_^2^ = 0.02
**Weight (kg)**	74.1±4.4	74.3±2.3	74±3.5	F (2;40) = 0.03; p = 0.97; η_p_^2^ = 0.002
**BMI (kg.m** ^**-2**^ **)**	24.1±1.8	24±1	24.1±1.2	F(2;40) = 0.003; p = 0.99; η_p_^2^ = 0.0002
**VO**_**2**_ **max (ml.min.kg** ^**-1**^ **)**	39±0.7	35.8±0.9***	34.3±0.8*** ###	F(2;40) = 100.11; p<0.001; η_p_^2^ = 0.87

BMI, body mass index; VO_2_max, maximum oxygen uptake;

***, significant differences compared to non-smokers at p < 0.001;

###, significant differences compared to cigarette smokers at p <0.001.

After the explanation of the nature and progress of the experimental protocol, an informed consent was signed by the subjects, as required by the Research Ethics Committee of the Faculty of Medicine, University of Sfax, Tunisia, and a physical fitness testing and medical examinations was established for each subject by our medical team. Participants were normolipidemic (fasting triglycerides < 1.7 mmol/L), nonobese. No subject used nutritional supplements or medications. Presence of any kind of disease or involvement in regular physical activity or exercise program during the previous 12 months was also exclusion criteria. On the basis of these criteria, 9 subjects from 52 were excluded. Eventually, 43 subjects were included in subsequent analysis and they were admitted to the training program.

Cigarette smokers and hookah were recruited according to the number of cigarettes and hookah per day and their career period. We consider a cigarette smoker any subject with consumption greater or equal to 10 pack-years (PY) and an average score of tobacco dependence of 8.12 ±1.41, measured by the Fagerström nicotine dependence test [40]. In the absence of specific international codification, we quantified hookah consumption, as in the study of Kiter et al. [41] in HY and kg of cumulative tobacco. The tobacco used in a hookah weighs between 10 and 25 g [42]. In fact, regular hookah smoker subjects are those having consumption greater or equal to 5 Hookah- years (HY) [43].

Participants were divided into three groups, and they performed a continuous training program 3 times a week for 12 weeks

A cigarette smokers group (CS) (n = 15); a hookah smokers group (HS) (n = 14) and another nonsmokers group (NS) (n = 14). All subjects underwent a test session and biochemical analyzes before and after the training program. The session includes anthropometric and physical tests, a biochemical analysis and antioxidant status review. All these measurements were performed by the same examiners to avoid methodological errors. An effort test was conducted before training program in order to quantify individual training loads.

### Anthropometric measurements

Body weight was measured to the nearest 100 g with a calibrated electronic scale (TANITA TBF.350 model), and height was measured to the nearest 1 mm with a fixed stadiometer.

### Blood Sampling and Biochemistry

Analyses were performed in the laboratory of Pharmacology of the Faculty of Medicine of Sfax. Smokers were instructed to refrain from smoking at least one hour prior to reporting to the laboratory as suggested by Dietrich et al. [44]. Blood samples were taken from an antecubital vein via needle, twice, before and after the training program. They were performed in basal conditions (8-am) after 12 hours fasting and 9 hours sleep. Blood was processed immediately and stored in microcentrifuge tubes at −80°C until analyzed. Total cholesterol (TC), triglycerides (TG), and high-density lipoprotein cholesterol (HDL-C) were measured in all subjects using the standardized techniques described by Wegge et al. [45] Low-density lipoprotein cholesterol (LDL-C) was calculated with the Friedewald formula [46]: [LDL = TC—HDL—(TG /2.18)]

All antioxidant markers were analyzed using commercially available assay kits, procured from Randox Laboratories (Randox Laboratories Ltd, placecountry-region UK). Plasma concentrations of superoxide dismutase (SOD), glutathione peroxidase (GPx), glutathione reductase (GR) and the total antioxidant status (TAS) were measured using standard colorimetric assays, and α-tocopherols was extracted with hexane from plasma and then measured via high performance liquid chromatography (HPLC). Malondialdehyde (MDA) was analyzed using malondialdehyde HPLC procedure. MDA concentrations were measured by the following formula: sample = (Peak height sample x concentration of the calibrator)/ Peak height calibrator.

### Aerobic fitness measurements

VO_2_max and maximal heart rate measurements during exercise were examined through treadmill maximal exercise test (COSMED Pulmonary-Function Equipment 37 Via dei Piani di monte Savello I-00040 Rome ITALY) using an analyzer (Fitmate PRO version 1.2 cosmed). This dynamic and maximum test, until fatigue, consists in increasing the speed of 1 km/h every 2 min, after warm up of 5 min with a 6 km/h speed until the participant could no longer continue. Heart rate was continually monitored throughout the exercise test using (Polar Electro Oy, Kempele, Finland). Verbal encouragement was provided throughout the test to ensure that a maximal effort was achieved.

### Dietary program

Participants were instructed to maintain their normal diet during the training period. Furthermore, we chose to standardize meal 72 hours before each blood sample. To do this, participants were instructed to be present in refectory of Sports Institute of Sfax each breakfast, lunch and dinner during the 72-hour period immediately preceding the blood samples (before and after training) in order to have similar dietary intake and avoid alterations in postprandial oxidative stress values.

### Continuous Training Protocol

Subjects of three groups underwent a continuous training program during a 3-months period. Training was performed continuously for 20 minutes (first month), 25 minutes (second month) and 30 minutes (third month), three times per week at an intensity of 40% of VO_2_max, on race track of 400 m at the Institute of Sport of Sfax, Tunisia. The cones placed and spaced 20 meters on a race track. At each beep, the subject must reach the following cone. All warm-ups before training should be between 50% and 60% of maximum heart rate for a period of about 10 minutes.

It was asked participants to run with a continuous rhythm respecting sound beeps and the requested time throughout the training session. The training load was insured by time and traveled distance and controlled by sound beeps. (T: the time between two cones; d: distance between two cones; V: proposed velocity).The load increase during the training period was provided by the increase in working time and the distance covered in each session. All participants successfully completed the training period and no recorded absences during all sessions. In addition, we have verified that there was no involvement in physical activity or exercise program throughout the 12-week training period.

### Statistical Analysis

Statistical analyses were performed using STATISTICA Software (StatSoft, France). The data were expressed as mean ± standard deviation (SD). After normality verification with the Shapiro-Wilk’s w test, and homogeneity of variances with Levene’s test, parametric tests were performed. One-way ANOVA was used to indicate inter group differences in the baseline subjects’ characteristics. Inter and intra-group comparisons of the variables were made by two-way ANOVA (group vs. training) with repeated measurements. Least Significant Different (LSD) post-hoc analysis was used to identify significant group differences that were indicated by one-way and two-way ANOVA. Effect sizes were calculated as partial eta-squared (η_p_^2^) to estimate the meaningfulness of significant findings. A p-value <0.05 was considered to be statistically significant.

## Results

Table 2 shows the values of antioxidants basal concentrations of smokers and nonsmokers.

**Table 2:** Antioxidant concentrations before training of 3 groups.

Parameters	Means±SD			ANOVA
	NS	CS	HS	
**GPx (U.gHg** ^**-1**^ **)**	26.77±1.67	25.99±2.86	26.68±2.07	F (2; 40) = 0.38; p = 0.69 ; η_p_^2^ = 0.02
**SOD (U.gHg** ^**-1**^ **)**	1024.2±41.4	1005.3±38.3	998.7±25.3	F (2; 40) = 1.5; p = 0.24 ; η_p_^2^ = 0.11
**DA (μmol.l** ^**-1**^ **)**	1.44±0.06	1.62±0.13***	1.59±0.12**	F (2; 40) = 9.12; p<0.001 ; η_p_^2^ = 0.49
**GR (U.gHg** ^**-1**^ **)**	7.41±0.93	6.6±0.5**	6.78±0.42*	F (2; 40) = 4.32; p = 0.023 ; η_p_^2^ = 0.21
**TAS (mmol.l** ^**-1**^ **)**	1.76±0.03	1.74±0.02	1.73±0.02*	F (2; 40) = 3.72; p = 0.036 ; η_p_^2^ = 0.17
**α-tocopherol (μmol)**	5.13±0.49	5.07±0.61	5.11±0.65	F (2; 40) = 0.03; p = 0.98 ; η_p_^2^ = 0.002

GPx, glutathione peroxidase; SOD, superoxide dismutase; MDA, malondialdehyde; GR, glutathione reductase; TAS, Total antioxidant status;

*, **, ***, Significant differences compared with nonsmokers at p <0.05, p<0.01, p<0.001 respectively.

The three groups had similar values of GPx, SOD and α-tocopherol. However, significant differences were observed for MDA, GR and TAS in smokers subjects compared to nonsmokers (p<0.001, p = 0.023 and p = 0.036, respectively). The post-hoc test revealed that before training, MDA of HS and CS groups were significantly superiors that of NS group (p<0.01 and p<0.001 respectively). The same for GR, a significant difference was observed between smokers and nonsmokers (p<0.01 for the CS group and p<0.05 for the HS group). Regarding TAS, The post-hoc test noted one significant difference between HS and NS groups (p<0.05).

### Continuous training effect on plasma antioxidants

The training period of 3 months induces a modification of all parameters of the antioxidant status of our subjects. However, this varies according to the group. We report in Table 2 the antioxidants improvement rate (∆) of 3 groups, observed in Pre vs. Post training program. In CS and HS groups, the SOD increase is significant; it is of the order of 9.6 ± 17.3 (U.gHg^-1^) and 9.6 ± 13.3 (U.gHg^-1^) respectively (p< 0.05) while it is only 5.7 ± 7.8 (U.gHg^-1^) in NS group (p>0.05). The training program induces also a significant increase in TAS in the three groups. This increase is more pronounced in CS group compared to that of HS and NS groups. It is respectively +0.05 ± 0.04 (μmol.l^-1^), +0.03 ± 0.04 (μmol.l^-1^) and +0.03 ± 0.04 (μmol.l^-1^) (p< 0.01, p < 0.05 and p< 0.05, respectively). Concerning MDA, the improvement is significant only in CS group (p<0.01). The α-tocopherol increase follows the same pattern. It is of the order of 2.49 ± 0.88 (μmol) (p< 0.05). The GR increase is significant only in CS and NS groups. It is respectively of +0.16 ± 0.18 (U.gHg^-1^) and +0.13 ± 0.26 (U.gHg^-1^). Finally, subjects in CS, HS and NS groups, showed GPx values increased. The increase is respectively 0.5 ± 0.76 (U.gHg^-1^), 0.44 ± 0.48 (U.gHg^-1^) (p< 0.05) and 0.65 ± 0.71 (U.gHg^-1^) (p< 0.01) (Table 3).

**Table 3:** Antioxidants improvement rate (∆) of the three groups after 12-week continuous training.

Parameters	Means±SD			Pre vs. Post		
	NS	CS	HS	NS	CS	HS
**GPx (U.gHg** ^**-1**^ **)**	0.65±0.71	0.5±0.76	0.44±0.48	††	†	†
**SOD (U.gHg** ^**-1**^ **)**	5.7±7.8	9.6±17.3	9.6±13.3		†	†
**MDA (μmol.l** ^**-1**^ **)**	-0.03±0.07	-0.11±0.17	-0.07±0.07		††	
**GR (U.gHg** ^**-1**^ **)**	0.13±0.26	0.16±0.18	0.05±0.12	†	†	
**TAS (mmol.l** ^**-1**^ **)**	0.03±0.04	0.05±0.04	0.03±0.04	†	††	†
**α-tocopherol (μmol)**	0.06±0.19	0.3±0.35	0.05±0.61		†	

NS, Nonsmokers; CS, Cigarette Smokers; HS, hookah smokers; GPx, Glutathione peroxidase; SOD, superoxide dismutase; MDA, Malondialdehyde; GR, Glutathione reductase; TAS: total antioxidant status.

†, ††, Significant Differences p<0.05, p<0.01, respectively.

### Continuous training effect on lipids and lipoproteins profile

The differences in the lipid improvement rate (Δ) of the three groups in pre vs. post program are summarized in Table 4. Our continuous training program induced, in most cases, significant improvements in plasma concentrations of the lipid and lipoprotein profile. The results show a significant increase in HDL-C in three groups. It is of the order of + 0.07 ± 0.08 (mmol.l^-1^) (p<0.01), + 0.08 ± 0.08 (mmol.l^-1^) (p<0.01) and + 0.05 ± 0.06 (mmol.l^-1^) (p <0.05) in NS, CS and HS respectively. In addition, a significant decrease in LDL-C was registered (NS: p<0.001, CS: p<0.001 and HS: p<0.01).

**Table 4:** Lipid improvement rate (Δ) of the three groups after 12-week continuous training.

Parameters	Means±SD			Pre vs. Post		
	NS	CS	HS	NS	CS	HS
**HDL-C (mmol.l** ^**-1**^ **)**	0.07±0.08	0.08±0.08	0.05±0.06	††	††	†
**LDL-C (mmol.l** ^**-1**^ **)**	-0.09±0.07	-0.12±0.09	-0.08±0.09	†††	†††	††
**TG (mmol.l** ^**-1**^ **)**	-0.01±0.03	-0.02±0.05	-0.01±0.05			
**TC (mmol.l** ^**-1**^ **)**	-0.02±0.03	-0.05±0.07	-0.04±0.04		††	†
**HDL-C/TG**	0.09±0.11	0.05±0.09	0.05±0.07	††		
**TC/HDL-C**	-0.32±0.29	-0.47±0.41	-0.34±0.36	††	†††	††

HDL-C, high-density lipoprotein cholesterol; LDL-C, low-density lipoprotein cholesterol; TC, total cholesterol; TG, triglycerides;

†, ††, †††, significant differences at p<0.05, p<0.01 and p<0.001 respectively.

Similarly, we recorded in these groups (NS, CS and HS) TC / HDL-C ratio reduced (p <0.01, p<0.001 and p<0.01 respectively). However, in smokers no significant change was recorded in HDL-C / TG ratio, while it was significantly higher after training program in nonsmokers group (+ 0.09 ± 0.11, p<0.01). In addition, all subjects had a reduction in total cholesterol (TC), but it was significant only for CS (p<0.01) and HS subjects (p<0.05). However, in these three groups no significant changes were recorded in TG.

## Discussion

As confirmed by many researchers who reported high levels of oxidative stress in smokers compared to nonsmokers [11,12], we found low basal antioxidant capacity in either cigarette and hookah smokers and have, therefore, important levels of oxidative stress compared to non-smokers (Table 2), A reasonable explanation for this finding may relate to the sedentary lifestyle of our participants. This indicates that a lower aerobic fitness level is associated with a Harmful higher oxidative stress level in smoker subjects, a finding that was described previously by Bloomer et al. [47].

Indeed, several studies have examined, using different protocols, the physical exercise or training effect on antioxidant status. However to our knowledge, no study has determined the independent contribution of continuous training on blood antioxidant markers in smoker male adults. For this, we chose to determine the contribution of 12-week low-intensity continuous training on the antioxidant defense capacity in sedentary cigarette and hookah smokers.

It is possible that randomized workouts can promote a lower antioxidant capacity and increased oxidative stress. However, according to Covas et al. [17], aerobic exercises could help to increase antioxidant defenses and reduce the oxidants production.

**Fig 1:**
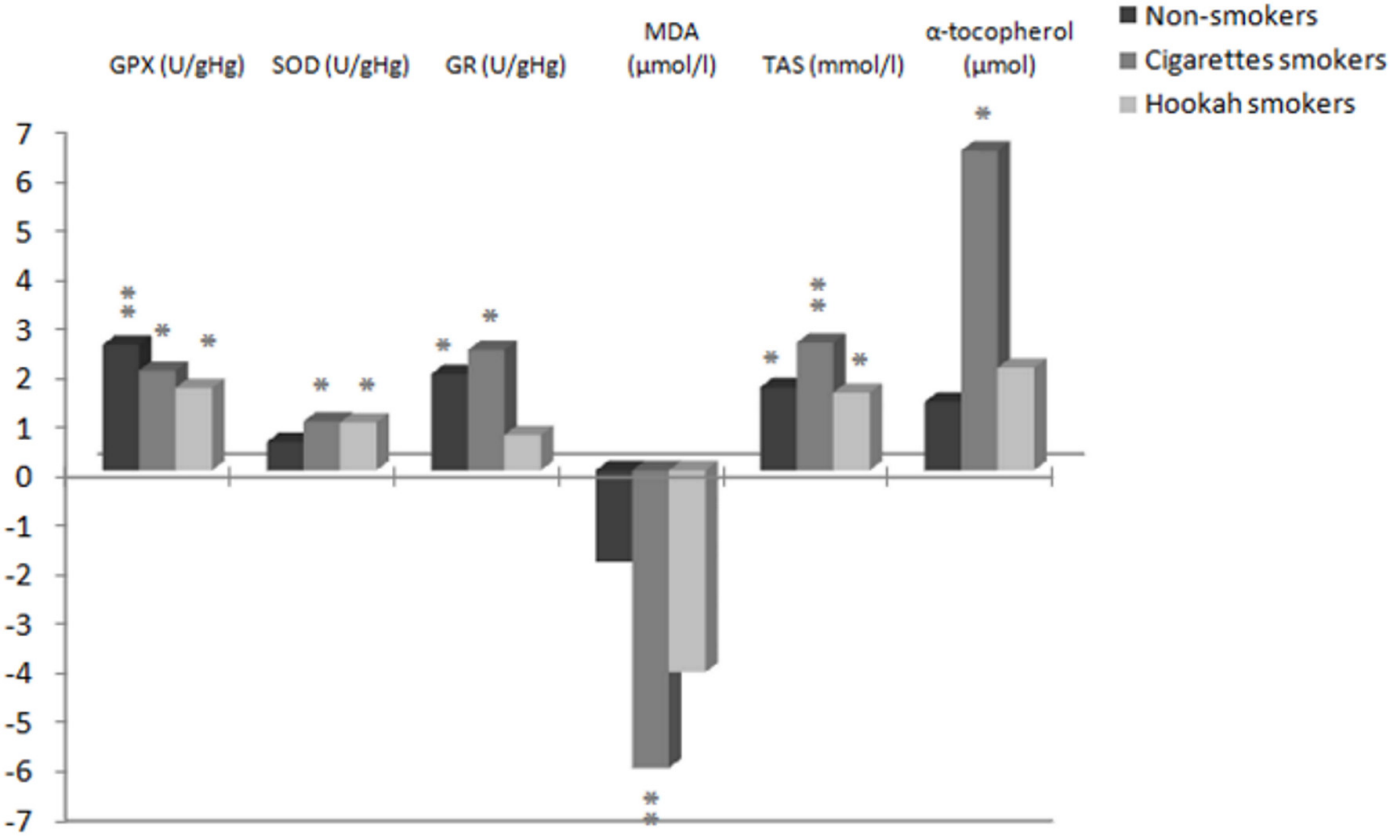
Antioxidants improvement rate in percentage of the three groups after training program. GPx, glutathione peroxidase; SOD, Superoxide dismutase; MDA, malondialdehyde; GR, glutathione reductase; TAS, total antioxydant status;*P<0.05; **P<0.01.

This experimental study showed improvements in antioxidants activity and lipid profile, and a decreased in MDA concentrations after 12-week continuous exercise aerobic training. These results are consistent with several other studies [23,48–50] that demonstrated an improvement in antioxidative status after short-term or long-term training. The study of Pialoux et al. [20] showed that 6-week endurance training reduced plasma MDA. Thus our continuous training program also led to a reduction of MDA in subjects of the three groups (from -2% to -6%) but was significant only for the CS group (p<0.01) (Fig 1).

Although aerobic training improves the antioxidant defense system in animal models [51,52], the effects of aerobic training on antioxidant activity in human are controversial. Higher levels of antioxidant activity have been observed in trained subjects than in sedentary ones [17,53]. Some previous studies report an increase of the antioxidant activity after training [23,24], while others have documented a decrease [26] or even no changes [25] in circulating antioxidants. This divergence could be explained in part, by the diversity of protocols implemented (Training methods, protocol duration, age of participants, smoking habits…) and the individual responses of each subject to exercise.

In this study, after 12-weeks low-intensity continuous training, the increase in GPx and SAT was statistically significant for three group subjects. The observed increase of GPx activity after the continuous training program has also been reported in two other studies [23,54]. The increase was approximately 2.6%, 2% and 1.7% for NS, CS and HS groups respectively. In addition, a significant increase in SOD was produced by continuous training in two smoker groups (p< 0.05). Although this parameter did not reach statistical significance in nonsmokers group, the difference was substantial (+5.7±7.8 U.gHg^-1^). The same effect was observed in an experimental study after leisure physical activity of low intensity [17]. Despite the higher physical activity level, the GR and α-tocopherol levels in HS group were unaltered after 12-weeks continuous training (+0.7% and +2% respectively). These findings are in contrast with several previous studies in human [54], and in animal [51,52] demonstrating an improvement in GR activity after training program. Several hypotheses could be advanced to explain this variety of intergroup response. The first ascertainment is that food intake is not the same from one subject to another. So, there are variable effects on oxidative stress. It seems that a diet rich in fat could make excessive reactions of oxidative stress. This idea was verified by Ma et al. [55], Palaniappan et al. [56], and Bloomer et al. [57]. The second ascertainment is that blood levels of antioxidants, before study, were not the same for cigarette and hookah smokers and the nonsmokers.

**Fig 2:**
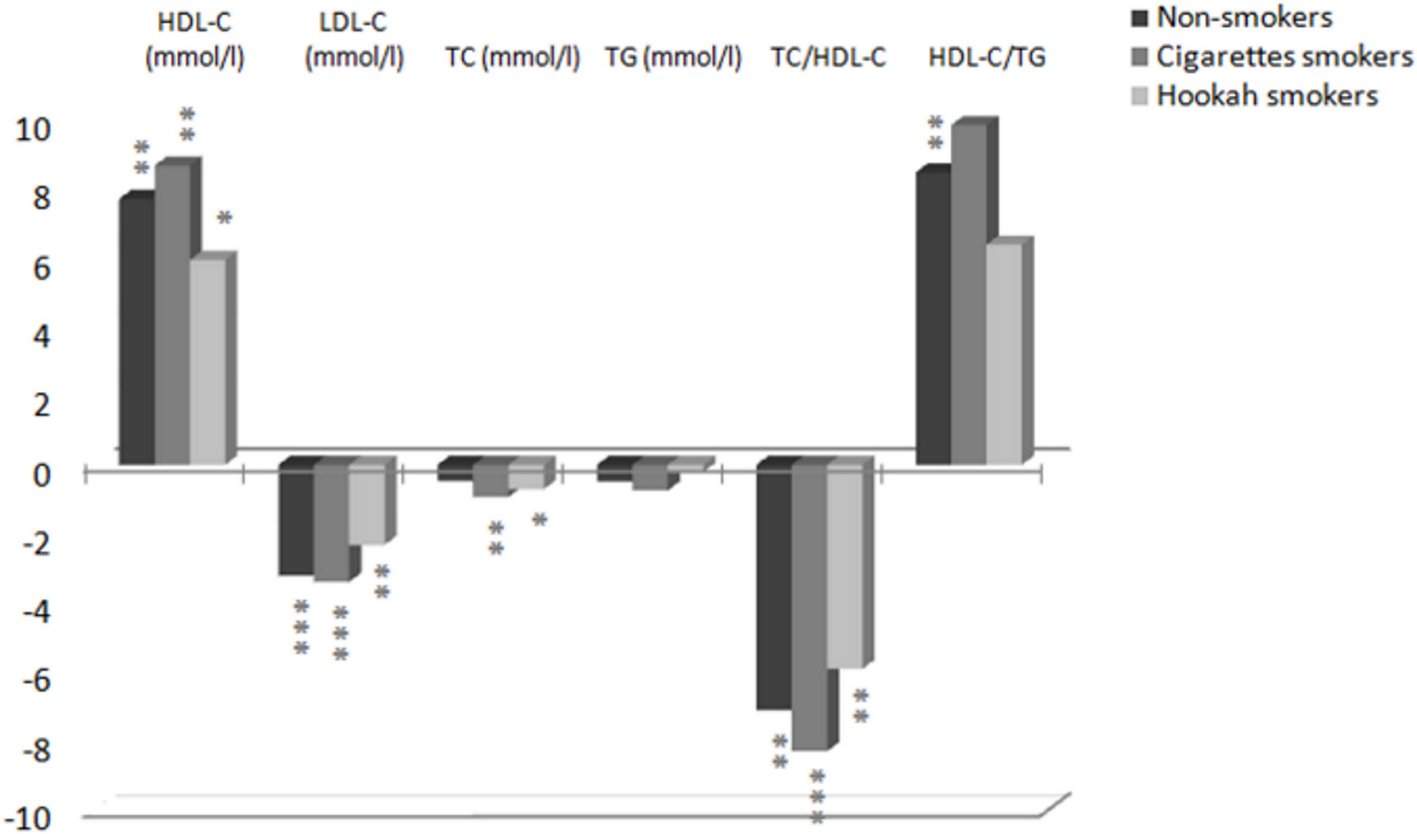
Lipids improvement rate in percentage of three groups after training program. HDL-C, high-density lipoprotein cholesterol; LDL-C, low-density lipoprotein cholesterol; TC, total cholesterol; TG, triglycerides, *P<0.05; **P<0.01; ***P<0.001.

After the low-intensity continuous training, we have found the TC/HDL-C ratio, which is considered to be a stronger predictor of coronary heart disease than HDL-C alone [58], decreased significantly more in the CS group (-8.3%), than in the NS and HS groups (-7.2% and -6% respectively) (Fig 2). These results are in agreement with the findings of Motoyama et al. [59] evaluating the effect of low intensity aerobic training on the lipid profile of elderly men and women. The authors demonstrated that TC, TG and LDL-C levels remained unaltered and significant increases in HDL-C and TC/HDL-C ratio.

In addition, our continuous training protocol has no significant effect on plasma TG for three groups. Similar results were reported by Bloomer et al. [60] in a cross-sectional study and by Tjonna et al. [61] which showed no change in TG value after a continuous training. Following this same training period, the high density lipoprotein-cholesterol (HDL-C) had increased significantly (ranging from 6% to 9%) in three group subjects. Our results are similar those reported by Donnelly et al. [62], which showed a change in HDL-C following a continuous training. These findings tend to be in line with previous observations of the effects of continuous low intensity exercise training on blood lipid profile [59]. However the TC had decreased significantly only in the smokers groups (p<0.01), but has not changed in the NS group. The HDL-C/ TG ratio had not changed significantly in either smoker group. As well, no significant difference was seen on serum lipids and lipoprotein concentrations between the smoker groups throughout the period. There was, however, a significant difference between CS and NS groups in TC (p<0.05) and between smoker groups and nonsmokers in HDL-C/ TG ratio, following 3 months of training (p<0.05).

Because the exercise intensity and training method have been adapted to the abilities of the participants, our continuous training program could be considered as an effective method to improve antioxidant status and lipid profile of smokers. The current study proposed a further demonstration concerning continuous training method which could be prescribed and recommended in smoker subjects.

Based on previous findings of Bloomer et al. [47] and the current study results, we suggest that training method with low-intensity continuous exercises can be considered as a "medicine" for cigarette and hookah smokers with increased risk of cardiovascular diseases and oxidative stress. Also, this training program seems to have a more transparent effect by associating with a dietary follow-up. This is indeed a limitation of this work, and should be considered relative to our findings.

## Conclusion

The findings of this study indicate that smokers have lower blood antioxidant capacity and higher blood lipid levels compared to nonsmokers. The smoking cessation is undoubtedly the best approach to reduce diseases caused by smoking, but the success rate among those trying to quit smoking is dismal. In contrast, the low-intensity continuous training is associated with improved of blood antioxidants and lipids and lipoproteins profile. Intensity and training volume have been continually monitored to demonstrate the contribution of continuous exercise, in reduction of oxidative stress in both cigarette and hookah smokers. People who are unable to quit smoking could focus at improving leisure time physical activity (by continuous exercises) in order to minimize some harm caused by smoking. Low-intensity continuous training appears to be beneficial and can be performed by sedentary adult smokers in order to mitigate some smoking harms.

### Limitations of the study

A limitation of the study is that diet during the training period was not controlled. However, study requires that participants follow the same diet in the 3 days preceding each blood sampling, and during the training period. The control group lack may be considered a limitation of the present study (smokers follow the same daily activity during the experimental protocol). Finally, our relatively small sample size could have limited our ability to detect group differences in our chosen markers. This is indeed a limitation of this work, and should be considered relative to our findings.
